# Smoking and vascular dysfunction in Africans and Caucasians from South Africa

**DOI:** 10.5830/CVJA-2010-034

**Published:** 2011-02

**Authors:** Mandlenkosi C Zatu, Johannes M Van Rooyen, Aletta E Schutte

**Affiliations:** Hypertension in Africa Research Team (HART), School for Physiology, Nutrition and Consumer Sciences, North-West University, Potchefstroom, South Africa; Hypertension in Africa Research Team (HART), School for Physiology, Nutrition and Consumer Sciences, North-West University, Potchefstroom, South Africa; Hypertension in Africa Research Team (HART), School for Physiology, Nutrition and Consumer Sciences, North-West University, Potchefstroom, South Africa

**Keywords:** smoking, vascular dysfunction, socio-economic status, ethnicity, Africans, Caucasians

## Abstract

**Background:**

Smoking is an important modifiable risk factor for cardiovascular disease, with limited research having been done in Africans. We aimed to determine the association between smoking and measurements of vascular function in Africans and Caucasians.

**Methods:**

We determined anthropometric and cardiovascular variables, serum cotinine and C-reactive protein (CRP) in African and Caucasian participants from South Africa (*n* = 630).

**Results:**

Africans had significantly lower body mass index (BMI), higher blood pressure and lower socio-economic status (SES) than Caucasians. Only African smokers showed increased arterial stiffness and a significant correlation between smoking and arterial stiffness. African smokers had increased and Caucasian smokers decreased high-density lipoprotein cholesterol (HDL-C) than the non-smokers. After adjusting for confounders, smoking showed few correlations, mainly with heart rate and CRP. In Africans, smoking also correlated positively with HDL-C, with the opposite result in Caucasians.

**Conclusion:**

African smokers had significantly increased arterial stiffness, which was not found in Caucasian smokers. Africans generally demonstrated more associations between smoking and cardiovascular dysfunction than Caucasians.

## Summary

It is well known that smoking has negative health consequences and it is the main avoidable cause of illness and death worldwide.^1^ Smoking causes many premature deaths annually in the world.^1^,^2^ Despite the negative effects of smoking, the continuous use of tobacco products is rising.^3^ Globally, the prevalence of smoking-related cardiovascular diseases (CVDs) is higher in Africans than Caucasians.^4^-^6^ In South Africa, factors such as age, gender, ethnicity, cultural and economic characteristics influence the prevalence of smoking.^7^,^8^ There is a high prevalence of smoking in adults, mostly white males and those earning a low income.^7^,^9^ However, it has been shown that poorer smokers are more likely to quit than smokers who are more affluent.^9^

Many studies have reported on the effect of smoking on the metabolic syndrome, which is a highly prevalent cluster of disorders that are relatively common in Africans.^10^-^12^ Smoking and the metabolic syndrome together cause dyslipidaemia, increased C-reactive protein (CRP) levels and endothelial dysfunction.^11^,^13^ Smokers are therefore characterised by high serum triglycerides (TG) and low-density lipoprotein cholesterol (LDL-C), with significantly lower high-density lipoprotein cholesterol (HDL-C) than non-smokers.^4^

Nicotine in cigarette smoke increases heart rate (HR) and cardiac output (CO) through cardiac beta-adrenergic effects,^1^ leading to increases in blood pressure (BP). Carbon monoxide decreases the oxygen-carrying capacity of the blood and may lead to ischaemia and hypoxia of the tissues.^4^,^14^ This stimulates increased red blood cell production, which contributes to increased viscosity and consequently inflammatory and coagulatory processes.^4^ Both inflammation and coagulation are associated with atherosclerosis and coronary heart disease.^15^,^16^ All these factors therefore contribute negatively, in one way or the other, to increased risk for CVD.

Nicotine is broken down metabolically into various metabolites that include cotinine and nicotine-N-oxide.^3^ The most important metabolite of nicotine is cotinine,^3^,^17^ which is a vital biological marker of smoking and has been used to identify smokers.^3^,^18^ Serum cotinine levels of smokers are consequently significantly higher than non-smokers.^3^

The association between smoking and CVD has been well documented in developed countries.^1^,^4^ However, limited data exist in low- and middle-income countries such as South Africa. The aim of this study was to determine if there are ethnic differences regarding the association between smoking and measures of cardiovascular function between African and Caucasian people of South Africa.

## Methods

This was a sub-study based on data from the SAfrEIC study (South African study on the influence of sex, age and Ethnicity on Insulin sensitivity and Cardiovascular function). The SAfrEIC study was a cross-sectional study with 630 participants (apparently healthy African and Caucasian men and women) from urban areas of the North West Province of South Africa, aged 20 to 70 years. Exclusion criteria for this sub-study were diabetes (type 1 and 2), or persons on diabetic medication, pregnant or breast-feeding women, and those testing positive for the human immunodeficiency virus (HIV).

The Ethics committee of the North-West University (Potchefstroom campus) approved this study. The participants signed informed consent forms after all procedures were explained to them. An interpreter was available to relay the information to the African subjects in their home language.

For a period of seven weeks, 10 to 20 participants visited the facility daily (consisting of 10 bedrooms, two bathrooms, a living room and kitchen) on the Potchefstroom campus of the North-West University. They arrived at 07:00 and four field workers accompanied the African participants, who were introduced to the setup. Each subject received a ‘participant sheet’, which guided him/her through the different research stations where the various measurements were done.

During the course of the morning, basic health, demographic and lifestyle questionnaires were completed. Participants were requested to indicate their income per month according to the codes in the questionnaire and they also had to specify the duration of smoking (in years) or use of tobacco products. A fasting blood sample was taken by a registered nurse from the antebrachial vein using sterile winged infusion sets and syringes, and anthropometric measurements were taken in a private room. Blood pressure (BP) and pulse-wave velocity (PWV) measurements were also taken in a private bedroom.

When all questionnaires were completed and all cardiovascular measurements taken, each participant received breakfast as well as a small financial compensation. In the event of a subject being identified with any abnormalities (such as hypertension or diabetes), the subject was referred to his/her local clinic, hospital or physician. Each subject received a short report containing his/her health information.

Height, body mass, waist circumference (WC) and hip circumference of each subject were taken according to standard procedures.^19^ The circumferences were measured in triplicate. Maximum height was measured to the nearest 0.1 cm using the Invicta Stadiometer (IP 1465, UK). Weight was measured to the nearest 0.1 kg using a digital scale (Precision Health Scale, A & D Co, Japan). A flexible metallic measuring tape was used to measure the circumferences, taken with the subjects standing upright, with the face directed towards the observer and the shoulders relaxed. The WC was measured at the thinnest visible point (below the last rib) of the trunk of the body. The hip circumference was measured at the broadest point over the gluteal muscles. Body mass index (BMI) was determined with the formula: body mass/body height^2^.

After a 10-minute rest in the sitting position, BP (systolic and diastolic) and HR were measured using the OMRON HEM-757 apparatus, with the BP cuff on the left upper arm. The appropriate cuff sizes were used for obese subjects. Two measurements were taken, with a five-minute rest interval.

PWV (both carotid-radialis and carotid-dorsalis pedis) was measured using the Complior SP apparatus. The following two distances were measured on the left side of each subject: carotidradialis (from the suprasternal notch to the radial artery in the wrist) and carotid-dorsalis pedis (from the suprasternal notch to the dorsalis pedis artery in the foot). The subtraction method was used, i.e. the distance from the carotid artery to the suprasternal notch was subtracted from the measurement to the dorsalis pedis or the radialis.

Cardiovascular parameters were monitored, making use of the Finometer™ device (FMS, Finapres Medical Systems, Amsterdam, Netherlands). This entailed a five-minute continuous recording of each subject’s cardiovascular parameters under resting, yet awake conditions. After the first two minutes the upper arm pressure was calibrated with the finger pressure for each individual subject (i.e. return-to-flow systolic calibration). The last two minutes of each recording were used to calculate the average of the cardiovascular variables, namely stroke volume (SV), cardiac output (CO), total peripheral resistance (TPR) and Windkessel arterial compliance (Cwk).

## Biochemical analyses

Plasma and serum samples were prepared using standard methods and stored at –80°C until analysis. High-sensitivity C-reactive protein (hs-CRP) and serum lipids were determined on a Konelab 20i (Labsystems Clinical Laboratory Division, Vantaa, Finland) clinical chemistry analyser. Cotinine analyses were performed using the IMMULITE 2000 nicotine metabolite assay (Siemens Medical Solutions Diagnostics Ltd, Los Angeles, CA, USA) and a solid-phase competitive chemiluminescent immunoassay (Catalog Number L2KNM6). HIV status was determined immediately after blood sampling with a rapid test, according to the protocol of the National Department of Health of South Africa. Serum was used for testing with the First Response test and was repeated with the Pareeshak test for confirmation.

## Statistical analysis

All statistical analyses were performed using Statistica version 8 (Statsoft, Inc, Tulsa, OK, 2007). Statistical results are presented as means, standard errors and 95% confidence intervals (CI). Variables that were not normally distributed were logarithmically transformed, namely TG and hs-CRP. An independent *t*-test and analysis of covariance (ANCOVA) were performed to compare the variables between the two ethnic groups and to determine significant differences. Self-reported smokers were included in the smoking group for statistical analyses.

Similar tests were performed to compare the variables between smokers and non-smokers within each ethnic group, and also while adjusting for age, gender, BMI and WC. Mean arterial pressure (MAP) was included as an adjustment variable while comparing PWV data. The Chi-square test was used to determine significant differences between categorical variables. We performed correlations and partial correlations between smoking and cardiometabolic values within each ethnic group. Complete datasets were not available for all participants during the statistical analysis, hence small discrepancies in participant numbers in the tables.

## Results

The characteristics of the African and Caucasian subject groups are compared in Table 1. Most variables differed significantly between the two groups (*p* ≤ 0.001). The African group had a higher proportion of smokers, whereas height, weight, BMI and WC levels were significantly higher in the Caucasians. Africans showed a more detrimental cardiovascular profile.

**Table 1.** Descriptive Statistics Of The Population Studied

*Variable*	*Africans (n = 258)*	*Caucasians (n = 372)*	p*-value*
Age (years)	41.6 ± 0.82 (39.9; 43.1)	40.4 ± 0.69 (39.0; 41.7)	0.287
Gender (men/women)	127/131	161/211	
Height (m)	1.63 ± 0.01 (1.62; 1.64)	1.72 ± 0.00 (1.71; 1.73)	≤ 0.001
Weight (kg)	63.5 ± 1.10 (61.3; 65.7)	82.2 ± 1.03 (80.2; 84.2)	≤ 0.001
BMI (kg/m^2^)	24.0 ± 0.45 (23.1; 24.9)	27.7 ± 0.31 (27.1; 28.3)	≤ 0.001
WC (cm)	78.7 ± 0.85 (77.0; 80.4)	87.4 ± 0.79 (85.8; 88.9)	≤ 0.001
SBP (mmHg)	127 ± 1.39 (124; 129)	119 ± 0.84 (118; 121)	≤ 0.001
DBP (mmHg)	85 ± 0.87 (83; 87)	78 ± 0.52 (77; 79)	≤ 0.001
HR (beats/min)	70.0 ± 0.82 (68.3; 72.0)	68.0 ± 0.50 (67.0; 68.4)	0.008
SV (ml)	75.2 ± 1.39 (72.5; 77.9)	90.8 ± 1.27 (88.4; 93.3)	≤ 0.001
CO (l/min)	4.99 ± 0.09 (4.81; 5.17)	6.12 ± 0.09 (5.94; 6.31)	≤ 0.001
TPR (mmHg.s/ml)	1.39 ± 0.04 (1.31; 1.46)	1.06 ± 0.02 (1.02; 1.09)	≤ 0.001
Cwk (ml/mmHg)	1.60 ± 0.03 (1.54; 1.67)	2.10 ± 0.03 (2.04; 2.17)	≤ 0.001
C-R PWV (m/s)	8.67 ± 0.10 (8.47; 8.87)	7.63 ± 0.07 (7.48; 7.77)	≤ 0.001
C-P PWV (m/s)	8.18 ± 0.10 (7.98; 8.38)	7.82 ± 0.06 (7.69; 7.94)	≤ 0.001
HDL-C (mmol/l)	1.57 ± 0.04 (1.49; 1.65)	1.39 ± 0.02 (1.35; 1.44)	≤ 0.001
LDL-C (mmol/l)	2.35 ± 0.06 (2.24; 2.46)	3.76 ± 0.07 (3.63; 3.89)	≤ 0.001
TG (mmmol/l)	1.38 ± 1.01 (1.36; 1.39)	1.46 ± 1.01 (1.44; 1.48)	≤ 0.001
hs-CRP (mg/l)	1.90 ± 1.03 (1.80; 2.01)	1.65 ± 1.02 (1.59; 1.72)	≤ 0.001
Smoking duration (years)	13.9 ± 0.82 (12.3; 15.5)	12.7 ± 1.46 (9.82; 15.7)	0.490
Cotinine (ng/ml)	199 ± 10.8 (178; 221)	43.2 ± 5.02 (33.3; 53.1)	≤ 0.001
*n* smokers (%)	161 (62.4)	54 (14.5)	≤ 0.001
*n* male smokers (%)	96 (76.0)	34 (21.1)	≤ 0.001
*n* female smokers (%)	65 (50.0)	20 (9.48)	≤ 0.001
Income/mth:			
≤ R1000	232 (90.0%)	23 (6.18%)	≤ 0.001
R1000–R2000	22 (8.52%)	29 (7.79%)	≤ 0.001
R2000–R3000	3 (1.16%)	14 (3.76%)	≤ 0.001
R3000–R4000	0 (0.00 %)	23 (6.18%)	≤ 0.001
R4000–R5000	1 (0.39%)	26 (6.99%)	≤ 0.001
≥ R5000	0 (0.00%)	244 (65.6%)	≤ 0.001

Values are expressed as the mean ± standard error (95% CI). The mean values for TG and hs-CRP were logarithmically transformed and geometric means used. BMI: body mass index; WC: waist circumference; SBP: systolic blood pressure; DBP: diastolic blood pressure; SV: stroke volume; CO: cardiac output; TPR: total peripheral resistance; Cwk: Windkessel compliance; C-R PWV: carotid-radialis pulse wave velocity; C-P PWV: carotid-dorsalis pedis pulse wave velocity; HDL-C: high-density lipoprotein cholesterol; LDL-C: low-density lipoprotein cholesterol; TG: triglycerides; hs-CRP: high-sensitivity C-reactive protein.

Caucasians had significantly lower hs-CRP values than the Africans, who presented a more favourable lipid profile than the Caucasians. Cotinine levels were significantly higher in Africans compared to their Caucasian counterparts, which was expected, due to higher numbers of reported smokers among the Africans. The results also revealed significant differences in socio-economic status (SES), in that the majority of African subjects were living on low incomes. A total of 90% of Africans earned less than R1 000 per month. By contrast, the majority of the Caucasian group (65.6%) was living on more than R5 000 per month.

Table 2 compares the African smokers and non-smokers. Smokers were older than non-smokers and had significantly lower weight, BMI and WC than non-smokers. Smokers showed significantly higher PWV, TG and cotinine levels than in non-smokers. CO was significantly higher in smokers than non-smokers in the African group. Both values of PWV, as a measure of arterial stiffness, were significantly higher in African smokers than non-smokers. Furthermore, HDL-C levels remained higher in African smokers than their non-smoking counterparts, even after adjusting for age, gender, BMI and WC.

**Table 2.** Comparison Between African Smokers And Non-Smokers

*Variable*	*African non-smokers (n = 93)*	*African smokers (n = 152)*	p*-value*
Age (years)	37.5 ± 1.36 (34.8; 40.2)	44.0 ± 0.98 (42.1; 46.0)	≤ 0.001
Gender (men/women)	31/66	96/65	
Height (m)	1.62 ± 0.01 (1.60; 1.63)	1.64 ± 0.01 (1.63; 1.65)	0.103
Weight (kg)	71.4 ± 1.99 (67.4; 75.4)	58.8 ± 1.15 (57.0; 61.0)	≤ 0.001
BMI (kg/m^2^)	27.4 ± 0.81 (25.8; 29.0)	22.1 ± 0.47 (21.1; 23.0)	≤ 0.001
WC (cm)	83.0 ± 1.54 (79.9; 86.0)	76.3 ± 0.95 (74.4; 78.2)	≤ 0.001
SBP (mmHg)	124 ± 2.64 (119; 129)	129 ± 1.55 (126; 132)	0.079
DBP (mmHg)	84 ± 1.19 (81.0; 87.2)	86 ± 1.02 (84.0; 88.0)	0.393
HR (beats/min)	69.3 ± 1.15 (67.0; 72.0)	70.3 ± 1.13 (68.0; 73.0)	0.561
SV (ml)	80.1 ± 1.58 (75.6; 85.0)	72.1 ± 1.72 (69.0; 75.5)	0.005
CO (l/min)	5.31 ± 0.16 (4.99; 5.63)	4.80 ± 0.12 (4.57; 5.00)	0.006
TPR (mmHg.s/ml)	1.28 ± 0.05 (1.18; 1.37)	1.46 ± 0.05 (1.36; 1.57)	0.016
Cwk (ml/mmHg)	1.76 ± 0.04 (1.66; 1.86)	1.50 ± 0.04 (1.42; 1.58)	≤ 0.001
C-R PWV (m/s)	8.05 ± 0.16 (7.76; 8.35)	9.04 ± 0.12 (8.79; 9.30)	≤ 0.001
C-P PWV (m/s)	7.57 ± 0.13 (7.25; 7.89)	8.55 ± 0.10 (8.31; 8.79)	≤ 0.001
HDL-C (mmol/l)	1.33 ± 0.04 (1.25; 1.41)	1.72 ± 0.06 (1.61; 1.84)	≤ 0.001
LDL-C (mmol/l)	2.36 ± 0.09 (2.18; 2.53)	2.35 ± 0.07 (2.21; 2.50)	0.962
TG (mmmol/l)	1.35 ± 1.01 (0.27; 0.32)	1.40 ± 1.01 (0.32; 0.35)	0.015
hs-CRP (mg/l)	1.95 ± 1.04 (0.59; 0.75)	1.88 ± 1.04 (0.55; 0.70)	0.434
Cotinine (ng/ml)	43.7 ± 9.13 (25.5; 61.8)	296 ± 10.9 (274; 317)	≤ 0.001
Comparison after adjustment for age, gender, BMI and WC			
SBP (mmHg)	128 ± 2.05 (124; 132)	126 ± 1.55 (123; 129)	0.050
DBP (mmHg)	85 ± 1.37 (82.1; 87.5)	85 ± 1.03 (82.8; 87.0)	0.443
HR (beats/min)	70.1 ± 1.38 (67.4; 73.0)	70.0 ± 1.04 (68.0; 72.0)	0.539
SV (ml)	74.5 ± 1.94 (70.7; 78.3)	75.2 ± 1.48 (72.3; 78.2)	≤ 0.001
CO (l/min)	4.91 ± 0.13 (4.66; 5.17)	5.01 ± 1.00 (4.81; 5.20)	≤ 0.001
TPR (mmHg.s/ml)	1.40 ± 0.06 (1.29; 1.51)	1.39 ± 0.04 (1.30; 1.47)	0.006
Cwk (ml/mmHg)	1.59 ± 0.03 (1.54; 1.65)	1.60 ± 0.02 (1.56; 1.65)	≤ 0.001
C-R PWV (m/s)	8.42 ± 0.15 (8.12; 8.72)	8.81 ± 0.11 (8.59; 9.03)	≤ 0.001
C-P PWV (m/s)	8.00 ± 0.12 (7.77; 8.23)	8.29 ± 0.09 (8.12; 8.47)	≤ 0.001
HDL-C (mmol/l)	1.44 ± 0.07 (1.31; 1.57)	1.64 ± 0.05 (1.54; 1.74)	≤ 0.001
LDL-C (mmol/l)	2.39 ± 0.09 (2.21; 2.58)	2.34 ± 0.07 (2.20; 2.48)	0.809
TG (mmmol/l)	1.35 ± 0.01 (0.27; 0.32)	1.40 ± 0.01 (0.32; 0.35)	0.012
hs-CRP (mg/l)	0.63 ± 0.04 (0.55; 0.71)	0.66 ± 0.03 (0.59; 0.72)	0.392
Cotinine (ng/ml)	53.9 ± 13.2 (27.8; 79.9)	288 ± 9.96 (269; 308)	≤ 0.001

Values are expressed as the mean ± standard error (95% CI). The mean values for TG and hs-CRP were logarithmically transformed and geometric means used. PWV was also adjusted for MAP. BMI: body mass index; WC: waist circumference; SBP: systolic blood pressure; DBP: diastolic blood pressure; SV: stroke volume; CO: cardiac output; TPR: total peripheral resistance; Cwk: Windkessel compliance; C-R PWV: carotis-radialis pulse wave velocity; C-P PWV: carotisdorsalis pedis pulse wave velocity; HDL-C: high-density lipoprotein cholesterol; LDL-C: low-density lipoprotein cholesterol; TG: triglycerides; hs-CRP: high-sensitivity C-reactive protein.

In Table 3 the Caucasian smokers and non-smokers are compared. There were fewer Caucasian smokers than non-smokers. HR and CO values were higher in the smokers, whereas TPR and Cwk values were lower in the smokers after adjustments were made. Smokers had lower HDL-C and higher TG and hs-CRP levels. Cotinine differed significantly throughout, with significantly higher values in smokers, as expected.

**Table 3.** Comparison Between Caucasian Smokers And Non-Smokers

*Variable*	*Caucasian non-smokers (n = 303)*	*Caucasian smokers (n = 53)*	p*-value*
Age (years)	41.4 ± 0.74 (39.9; 42.8)	35.0 ± 1.67 (31.6; 38.2)	≤ 0.001
Gender (men/women)	127/191	34/20	
Height (m)	1.71 ± 0.01 (1.71; 1.73)	1.73 ± 0.01 (1.70; 1.75)	0.444
Weight (kg)	82.0 ± 1.12 (79.8; 84.1)	84.1 ± 2.75 (78.6; 89.6)	0.453
BMI (kg/m^2^)	27.7 ± 0.34 (27.0; 28.4)	28.0 ± 0.81 (26.4; 29.7)	0.686
WC (cm)	87.2 ± 0.85 (85.5; 88.9)	89.0 ± 2.19 (84.6; 93.4)	0.410
SBP (mmHg)	119 ± 0.93 (118; 121)	119 ± 1.98 (115; 123)	0.909
DBP (mmHg)	78 ± 0.56 (77.1; 79.3)	79 ± 1.42 (75.6; 81.3)	0.863
HR (beats/min)	67.0 ± 0.53 (66.0; 68.1)	71.0 ± 1.24 (68.0; 73.0)	0.014
SV (ml)	90.3 ± 1.36 (87.6; 93.0)	94.1 ± 3.43 (87.3; 101)	0.283
CO (l/min)	6.04 ± 0.10 (5.85; 6.24)	6.57 ± 0.28 (6.01; 7.14)	0.046
TPR (mmHg.s/ml)	1.07 ± 0.02 (1.03; 1.11)	0.96 ± 0.04 (0.89; 1.04)	0.027
Cwk (ml/mmHg)	2.07 ± 0.03 (2.00; 2.13)	2.33 ± 0.08 (2.17; 2.48)	0.002
C-R PWV (m/s)	7.62 ± 0.08 (7.46; 7.78)	7.62 ± 0.14 (7.34; 7.89)	0.982
C-P PWV (m/s)	7.82 ± 0.07 (7.69; 7.95)	7.82 ± 0.15 (7.52; 8.12)	0.983
HDL-C (mmol/l)	1.42 ± 0.02 (1.37; 1.47)	1.22 ± 0.06 (1.10; 1.33)	≤ 0.001
LDL-C (mmol/l)	3.76 ± 0.07 (3.62; 3.90)	3.80 ± 0.16 (3.47; 4.13)	0.837
TG (mmol/l)	1.45 ± 0.01 (0.36; 0.39)	1.54 ± 0.02 (0.39; 0.47)	0.005
hs-CRP (mg/l)	1.63 ± 0.02 (0.45; 0.53)	1.80 ± 0.05 (0.49; 0.69)	0.060
Cotinine (ng/ml)	10.4 ± 0.78 (8.89; 11.9)	231 ± 18.5 (194; 268)	≤ 0.001
Comparison after adjustment for age, gender, BMI and WC			
SBP (mmHg)	119 ± 0.74 (118; 121)	120 ± 1.84 (116; 123)	0.970
DBP (mmHg)	78 ± 0.46 (77.1; 78.9)	79 ± 1.14 (77.1; 81.6)	79 ± 1.14 (77.1; 81.6)
HR (beats/min)	67.0 ± 0.52 (66.0; 68.0)	71.0 ± 1.29 (68.2; 73.2)	0.014
SV (ml)	91.2 ± 1.04 (89.1; 93.2)	89.3 ± 2.59 (84.2; 94.4)	0.165
CO (l/min)	6.11 ± 0.08 (5.95; 6.26)	6.26 ± 0.19 (5.87; 6.64)	0.012
TPR (mmHg.s/ml)	1.06 ± 0.02 (1.03; 1.09)	1.04 ± 0.04 (0.96; 1.12)	0.011
Cwk (ml/mmHg)	2.11 ± 0.02 (2.08; 2.14)	2.05 ± 0.04 (1.97; 2.13)	≤ 0.001
C-R PWV (m/s)	7.62 ± 0.07 (7.48; 7.76)	7.65 ± 0.18 (7.29; 8.01)	0.423
C-P PWV (m/s)	7.79 ± 0.05 (7.69; 7.90)	7.98 ± 0.14 (7.71; 8.25)	0.423
HDL-C (mmol/l)	1.41 ± 0.02 (1.37; 1.45)	1.29 ± 0.05 (1.20; 1.39)	≤ 0.001
LDL-C (mmol/l)	3.75 ± 0.07 (3.62; 3.88)	3.89 ± 0.17 (3.57; 4.22)	0.859
TG (mmol/l)	1.45 ± 0.01 (0.36; 0.38)	1.54 ± 0.02 (0.40; 0.47)	0.002
hs-CRP (mg/l)	1.62 ± 0.02 (0.45; 0.52)	1.84 ± 0.04 (0.52; 0.70)	0.029
Cotinine (ng/ml)	10.2 ± 3.06 (4.23; 16.3)	232 ± 7.47 (218; 247)	≤ 0.001

Values are expressed as the mean ± standard error (95% CI). The mean values for TG and hs-CRP were logarithmically transformed and geometric means used. PWV was also adjusted for MAP. BMI: body mass index; WC: waist circumference; SBP: systolic blood pressure; DBP: diastolic blood pressure; SV: stroke volume; CO: cardiac output; TPR: total peripheral resistance; Cwk: Windkessel compliance; C-R PWV: carotid-radialis pulse wave velocity; C-P PWV: carotiddorsalis pedis pulse wave velocity; HDL-C: high-density lipoprotein cholesterol; LDL-C: low-density lipoprotein cholesterol; TG: triglycerides; hs-CRP: high-sensitivity C-reactive protein.

Further analyses were performed in which smoking was correlated with cardiovascular variables (SBP, DBP, HR, CO, Cwk, PWV), hs-CRP and lipid (HDL-C, LDL-C, TG) levels. To correlate smoking with these variables, smoking was viewed as either chronic exposure, using the subjects’ duration of smoking (obtained from questionnaires), or acute exposure, using serum cotinine values.

**Fig. 1.**
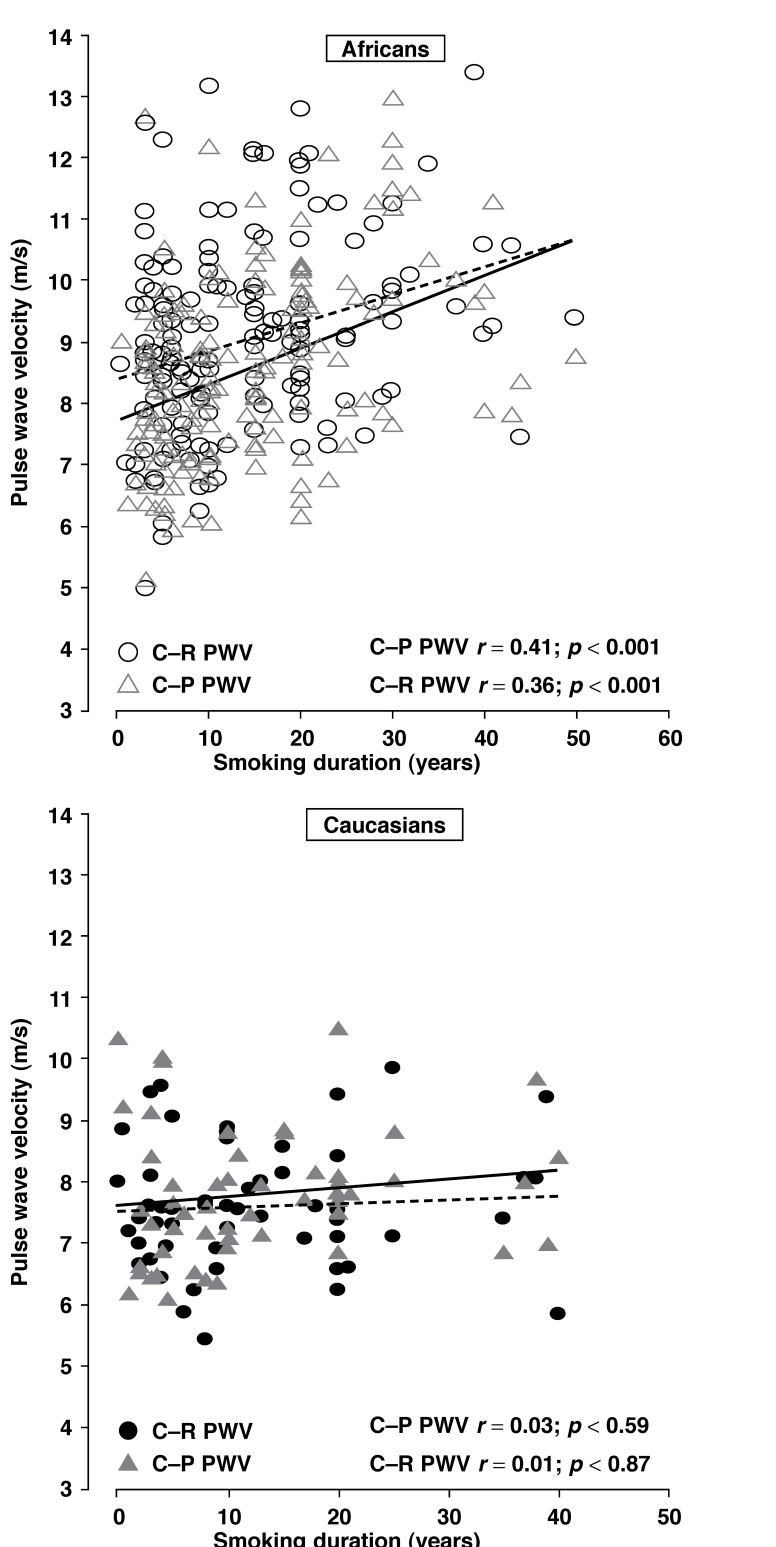
The relationship between duration of smoking and arterial stiffness in Africans and Caucasians. C-R PWV: carotid-radialis pulse wave velocity; C-P PWV: carotiddorsalis pedis pulse wave velocity.

Table 4 correlates smoking with the above variables in Africans. The results showed that chronic exposure to smoking (smoking duration) had significant correlations with most cardiovascular variables in the whole African group before adjustments were made. This trend remained quite similar after dividing the African group according to gender. PWV (Fig. 1), CO and Cwk also showed strong, significant correlations with smoking duration (*p* ≤ 0.001).

**Table 4.** Correlations Between Smoking And Measures Of Cardiovascular Function And Lipids In African Participants

*Variable*	*Men (n = 127)*				*Women (n = 131)*				*Whole group (n =258)*			
	*Smoking duration*		*Cotinine*		*Smoking duration*		*Cotinine*		*Smoking duration*		*Cotinine*	
	r*-value*	p*-value*	r*-value*	p*-value*	r*-value*	p*-value*	r*-value*	p*-value*	r*-value*	p*-value*	r*-value*	p*-value*
SBP (mmHg)	0.22	0.012	–0.05	0.593	0.17	0.043	0.02	0.795	0.23	≤ 0.001	0.03	0.680
DBP (mmHg)	0.21	0.019	–0.04	0.678	0.18	0.039	0.03	0.750	0.17	0.006	–0.08	0.901
HR (beats/min)	0.34	≤ 0.001	0.07	0.428	0.74	0.403	–0.05	0.586	0.18	0.004	–0.02	0.727
CO (l/min)	–0.13	0.141	–0.12	0.183	–0.28	≤ 0.001	–0.18	0.046	–0.20	≤ 0.001	–0.16	0.011
TPR (mmHg.s/ml)	0.15	0.104	0.13	0.153	0.33	≤ 0.001	0.15	0.096	0.21	≤ 0.001	0.01	0.024
Cwk (ml/mmHg)	–0.54	≤ 0.001	–0.19	0.035	–0.40	≤ 0.001	–0.23	0.008	–0.40	≤ 0.001	–0.17	0.006
C-R PWV (m/s)	0.36	≤ 0.001	0.15	0.102	0.22	0.012	0.30	≤ 0.001	0.36	≤ 0.001	0.27	≤ 0.001
C-P PWV (m/s)	0.46	≤ 0.001	0.16	0.077	0.24	0.005	0.26	0.002	0.41	≤ 0.001	0.25	≤ 0.001
HDL-C (mmol/l)	0.25	0.005	0.19	0.039	0.21	0.019	0.08	0.352	0.28	≤ 0.001	0.18	0.005
LDL-C (mmol/l)	0.08	0.400	0.04	0.641	0.12	0.162	0.12	0.186	0.35	0.578	0.04	0.502
TG (mmmol/l)	0.04	0.684	–0.00	0.995	0.21	0.016	0.22	0.011	0.74	0.239	0.11	0.096
hs-CRP (mg/l)	0.24	0.006	0.09	0.338	0.05	0.556	0.00	0.957	0.95	0.132	0.01	0.932
Cotinine (ng/ml)	0.44	≤ 0.001	–	–	0.49	≤ 0.001	–	–	0.48	≤ 0.001	–	–
After adjustment for age, BMI and WC												
SBP (mmHg)	0.08	0.357	–0.03	0.753	–0.04	0.624	–0.13	0.132	–0.00	0.968	–0.10	0.129
DBP (mmHg)	0.12	0.185	0.03	0.757	0.06	0.476	–0.05	0.589	0.07	0.270	–0.04	0.526
HR (beats/min)	0.13	0.153	–0.03	0.706	–0.00	0.996	–0.12	0.190	0.13	0.047	–0.08	0.202
CO (l/min)	–0.01	0.933	0.04	0.696	–0.09	0.317	0.01	0.950	0.02	0.788	0.03	0.588
TPR (mmHg.s/ml)	–0.00	0.963	0.07	0.432	0.10	0.258	–0.05	0.591	–0.02	0.812	–0.00	0.965
Cwk (ml/mmHg)	–0.16	0.075	0.05	0.588	–0.06	0.535	0.09	0.329	–0.10	0.119	0.11	0.095
C-R PWV (m/s)	0.05	0.656	0.01	0.911	0.09	0.444	0.14	0.279	0.15	0.071	0.07	0.408
C-P PWV (m/s)	0.06	0.585	–0.06	0.567	–0.17	0.199	0.29	0.025	0.04	0.588	0.09	0.246
HDL-C (mmol/l)	0.14	0.135	0.02	0.851	0.05	0.575	–0.05	0.551	0.13	0.035	0.04	0.523
LDL-C (mmol/l)	–0.02	0.810	0.07	0.441	0.05	0.607	0.07	0.441	–0.01	0.880	0.05	0.444
TG (mmmol/l)	–0.02	0.860	0.11	0.220	0.08	0.382	0.14	0.117	–0.02	0.811	0.12	0.062
hs-CRP (mg/l)	0.05	0.565	0.03	0.731	0.11	0.224	0.07	0.473	0.13	0.038	0.08	0.187
Cotinine (ng/ml)	0.31	≤ 0.001	–	–	0.40	≤ 0.001	–	–	0.35	≤ 0.001	–	–

PWV was additionally adjusted for MAP. SBP: systolic blood pressure; DBP: diastolic blood pressure; HR: heart rate; CO: cardiac output; TPR: total peripheral resistance; Cwk: Windkessel compliance; C-R PWV: carotid-radialis pulse wave velocity; C-P PWV: carotid-dorsalis pedis pulse wave velocity; HDL-C, high-density lipoprotein cholesterol; LDL-C, low-density lipoprotein cholesterol; TG, triglycerides; hs-CRP, high-sensitivity C-reactive protein; MAP, mean arterial pressure.

The relationships between cotinine levels and cardiovascular variables were somewhat weaker in the whole African group. A positive correlation was found between cotinine and PWV, and weak correlations with TPR and Cwk. HDL-C levels correlated positively and significantly with smoking in the African group. On adjustment for age, BMI and WC, almost all correlations became weak and non-significant. HR remained significant when correlated with chronic smoking. Hs-CRP correlated weakly with increased chronic exposure in the whole group of Africans, while cotinine levels correlated positively with smoking duration in all cases in the African group.

The Caucasian group (Table 5) generally did not show strong correlations with smoking (both chronic and acute). However, CO (*r* = 0.13), TPR (*r* = –0.12) and Cwk (*r* = 0.13) did reflect statistically significant correlations with cotinine levels in the whole Caucasian group. Most correlations disappeared after adjusting for age, BMI and WC. A significant correlation could also be seen between cotinine levels and the duration of smoking in the Caucasian group. Moreover, HR correlated positively and significantly with smoking throughout the Caucasian group.

**Table 5.** Correlations Between Smoking And Measures Of Cardiovascular Function And Lipids In Caucasian Participants

*Variables*	*Men (n = 161)*				*Women (n = 211)*				*Whole group (n = 372)*			
	*Smoking duration*		*Cotinine*		*Smoking duration*		*Cotinine*		*Smoking duration*		*Cotinine*	
	r*-value*	p*-value*	r*-value*	p*-value*	r*-value*	p*-value*	r*-value*	p*-value*	r*-value*	p*-value*	r*-value*	p*-value*
SBP (mmHg)	0.05	0.556	–0.07	0.397	–0.03	0.694	–0.05	0.454	0.07	0.161	–0.01	0.889
DBP (mmHg)	0.04	0.597	–0.05	0.558	0.13	0.061	0.06	0.362	0.09	0.078	0.03	0.565
HR (beats/min)	0.18	0.021	0.18	0.023	0.20	0.004	0.17	0.019	0.15	0.005	0.15	0.004
CO (l/min)	0.03	0.751	0.09	0.226	0.10	0.137	0.11	0.118	0.09	0.095	0.13	0.013
TPR (mmHg.s/ml)	0.00	0.989	–0.10	0.199	–0.06	0.426	–0.09	0.156	–0.05	0.329	–0.12	0.019
Cwk (ml/mmHg)	–0.13	0.098	0.07	0.410	0.02	0.762	0.06	0.364	0.01	0.786	0.13	0.016
C-R PWV (m/s)	0.09	0.279	–0.12	0.154	0.05	0.444	0.04	0.588	0.01	0.870	–0.01	0.902
C-P PWV (m/s)	–0.01	0.902	–0.02	0.770	0.00	0.956	–0.02	0.779	0.03	0.558	0.01	0.921
HDL-C (mmol/l)	–0.16	0.046	–0.20	0.014	–0.03	0.702	–0.00	0.945	–0.17	≤ 0.001	–0.15	0.004
LDL-C (mmol/l)	0.07	0.379	0.06	0.493	0.03	0.716	–0.04	0.617	0.06	0.286	0.02	0.737
TG (mmmol/l)	0.15	0.052	0.10	0.194	0.08	0.237	–0.00	0.974	0.16	0.003	0.08	0.116
hs-CRP (mg/l)	0.14	0.073	0.14	0.087	0.09	0.191	0.07	0.303	0.09	0.102	0.08	0.113
Cotinine (ng/ml)	0.71	≤ 0.001	–	–	0.84	≤ 0.001	–	–	0.74	≤ 0.001	–	–
After adjustment for age, BMI and WC												
SBP (mmHg)	0.02	0.854	–0.04	0.660	–0.00	0.970	0.01	0.924	–0.00	0.957	–0.01	0.813
DBP (mmHg)	0.14	0.863	–0.02	0.844	0.18	0.013	0.14	0.050	0.07	0.198	0.06	0.240
HR (beats/min)	0.20	0.016	0.17	0.033	0.20	0.006	0.17	0.023	0.18	≤ 0.001	0.17	0.002
CO (l/min)	0.09	0.296	0.06	0.482	0.13	0.066	0.16	0.021	0.09	0.102	0.11	0.044
TPR (mmHg.s/ml)	–0.07	0.406	–0.05	0.569	–0.05	0.477	–0.11	0.138	–0.05	0.327	–0.07	0.161
Cwk (ml/mmHg)	–0.07	0.364	–0.08	0.300	–0.02	0.807	0.22	0.755	–0.07	0.216	–0.02	0.649
C-R PWV (m/s)	–0.18	0.349	–0.23	0.226	–0.45	0.096	0.22	0.438	–0.27	0.056	0.02	0.901
C-P PWV (m/s)	–0.00	0.980	–0.11	0.557	–0.03	0.925	0.39	0.156	–0.11	0.457	0.10	0.490
HDL-C (mmol/l)	–0.20	0.012	–0.18	0.027	–0.03	0.652	–0.02	0.395	–0.12	0.026	–0.10	0.052
LDL-C (mmol/l)	0.06	0.470	0.07	0.393	0.04	0.539	0.00	0.985	0.04	0.413	0.04	0.459
TG (mmmol/l)	0.16	0.050	0.12	0.131	0.11	0.111	0.05	0.503	0.13	0.012	0.09	0.088
hs-CRP (mg/l)	0.17	0.037	0.14	0.096	0.11	0.106	0.12	0.090	0.13	0.015	0.13	0.017
Cotinine (ng/ml)	0.73	≤ 0.001	–	–	0.84	≤ 0.001	–	–	0.74	≤ 0.001	–	–

PWV was additionally adjusted for MAP. SBP: systolic blood pressure; DBP: diastolic blood pressure; HR: heart rate; CO: cardiac output; TPR: total peripheral resistance; Cwk: Windkessel compliance; C-R PWV: carotid-radialis pulse wave velocity; C-P PWV: carotid-dorsalis pedis pulse wave velocity; HDL-C: high-density lipoprotein cholesterol; LDL-C: low-density lipoprotein cholesterol; TG: triglycerides; hs-CRP: high-sensitivity C-reactive protein; MAP: mean arterial pressure.

A statistically significant negative correlation existed between smoking (both chronic and acute) and HDL-C levels before and after adjustments. TG values increased with chronic exposure to smoking, even after adjusting for age, BMI and WC. Hs-CRP levels correlated positively and significantly with smoking duration, but only after adjustment for age and obesity markers (BMI, WC).

## Discussion

Smoking is associated with vascular dysfunction.^1^,^20^,^21^ Since studies regarding this topic are limited in South Africa, the objective was to investigate ethnic differences with regard to the association between smoking and cardiometabolic markers. The cardiovascular markers related to smoking were generally higher in Africans (BP, PWV, hs-CRP and cotinine) – a finding supported by the literature.^9^,^22^

Immense differences in SES between the two groups could be the contributing factor in this regard. Cheaper mentholated tobacco brands used by Africans had more nicotine, whereas the non-mentholated, lighter brands smoked by the Caucasians had less effect on vascular function.^5^ Our results further revealed that the African smokers were older than the non-smokers, weighed less, and had lower BMI and WC values. Nicotine accelerates lipid breakdown^1^ and this may lead to weight loss in smokers. Some smokers use tobacco smoke for this purpose,^23^,^24^ and urbanised South African women are no exception.^25^ The LDL-C and TG levels were higher in Caucasians compared to Africans, a feature supported by the literature.^26^,^27^

The differences between smokers and non-smokers regarding cardiovascular risk factors were also investigated in both ethnicities. Significant differences were observed and were in most cases consistent with findings in the literature.^21^,^28^ Regular cigarette smoking increases HR and CO acutely throughout the day,^1^ a finding evident especially in the Caucasian smokers. The increases in HR and CO are mediated by the beta-adrenergic effects of nicotine.^1^ Although nicotine does constrict some peripheral vascular beds,^4^ it is likely that with increased HR and CO, nicotine appears to dilate other vascular beds through stimulation of epinephrine release,^1^,^4^ thereby decreasing TPR, as was found in our study groups.

A finding of this study that was not consistent with the literature was higher HDL-C levels in African smokers, and this result is in direct contrast to the significantly lower HDL-C values in Caucasian smokers. Furthermore, HDL-C values correlated positively with smoking in Africans, and negatively in Caucasians. The literature is sparse regarding this finding, although increased HDL-C levels in smoking Africans with cardiovascular disease have been mentioned.^28^,^29^ The high HDL-C levels possibly serve as a defense mechanism in Africans against the oxidative stress caused by smoking, as they are generally known to have higher HDL-C levels than Caucasians.^26^

Moreover, the infrequent use of cigarettes and more use of snuff in Africans with lower SES than in Caucasians has revealed no change in HDL-C values between smokers and non-smokers.^30^-^32^ This is a finding that is likely to apply in our African participants, with consequently less HDL-C destruction by oxidant gases from smoke. However, a higher HDL-C level in African smokers than in their non-smoker counterparts is a finding that needs further research for more clarity.

Smoking was also correlated with other cardiometabolic variables and, in comparison to previous findings,^1^,^4^,^13^ smoking correlated poorly with the measured variables, especially after adjustment for confounders. PWV, HDL-C and hs-CRP were the only variables with stronger correlations observed in Africans.

The generally weak correlations in the study may be due to the relatively young age of the smokers, with stronger correlations only expected at older ages. Weak correlations may also be affected by misclassification of chronic smoking by participants, as it was determined by self-reporting only,^33^ especially in Africans, who may suspend smoking on financial grounds.^24^ The weak correlations in Caucasians may be explained by the less nicotine-packed brands they use and their generally lower arterial stiffness and TPR compared to Africans.^6^,^26^ The higher cotinine levels of Caucasians may be explained by the generally low clearance rate of cotinine in Caucasians.^3^

The higher TG levels of the smokers in this study is a finding consistent with the literature.^1^,^4^,^29^,^34^,^35^ Increased TG levels are due to nicotine-mediated lipolysis and also increased corticosteroid and growth hormone levels, inducing insulin resistance,^1^,^4^ especially in smokers who are also snuff users.^1^

Hs-CRP, a well-known inflammatory marker,^36^ correlated positively with smoking duration in both groups, and with acute smoking in Caucasians (Table 5). Endothelial dysfunction caused by smoking promotes inflammation that leads to increased hs-CRP levels in smokers.^35^,^37^ Age, gender, ethnicity, obesity, chronic infections and low SES are responsible for variations in hs-CRP levels.^38^ As a result, the concentration of hs-CRP between smokers and non-smokers may be masked. This is reflected to some extent in this study, where hs-CRP levels showed weak correlations with acute smoking throughout and also in the women participants.

## Study limitations

Limitations in scientific research are difficult to avoid, and this study is no exception. Firstly, it was not possible to match the two ethnic groups for SES. Secondly, the study made use of volunteers, and subjects were therefore not selected on a random basis. This sampling method may also have influenced the difference in the number of smokers in the two ethnic groups. Thirdly, coagulation markers did not form part of this study, especially since it is known that Africans suffer from very high levels of fibrinogen,^20^ and since smoking plays an important role with regard to coagulation.^1^,^4^

## Conclusion

African smokers had significantly increased arterial stiffness, which was not found in the Caucasian smokers. Africans also showed more associations between smoking and cardiovascular dysfunction than the Caucasians. A high degree of urbanisation among Africans,^39^ coupled with higher smoking prevalence might be to blame for the high prevalence of cardiovascular diseases in the African population.
